# Hip fracture fixation in a patient with below-knee amputation presents a surgical dilemma: a case report

**DOI:** 10.1186/1752-1947-2-296

**Published:** 2008-09-09

**Authors:** Ulfin Rethnam, Rajam Sheeja Yesupalan, Amer Shoaib, Thanga K Ratnam

**Affiliations:** 1 Department of Orthopaedics, Glan Clwyd Hospital, Bodelwyddan LL18 5UJ, UK; 2 Department of Orthopaedics, Stepping Hill hospital, Stockport, UK; 3 Department of Orthopaedics, Wrexham Maelor hospital, Wrexham, UK

## Abstract

**Introduction:**

Hip fracture fixation surgery in patients with below-knee amputations poses a challenging problem to the surgeon in terms of obtaining traction for reduction of the fracture. The absence of the foot and part of the leg in these patients makes positioning on the fracture table difficult. We highlight this difficult problem and suggest techniques to overcome it.

**Case presentation:**

A 73-year-old man with bilateral below-knee amputations presented with a history of fall. Radiographs revealed an inter-trochanteric fracture of the femur. A dynamic hip screw fixation was planned for the fracture but the dilemma was on how to position the patient on the fracture table for the surgery. Special attention was needed in positioning the patient and in surgical fixation of the fracture.

**Conclusion:**

Hip fracture fixation in patients with below-knee amputations poses a special problem in positioning for fracture reduction and fixation. In this case report, we share our experience and suggest techniques to use when encountering this difficult problem.

## Introduction

Inter-trochanteric fractures have traditionally been treated by closed reduction and internal fixation with a dynamic hip screw or an intramedullary device (gamma nail, reconstruction nail, proximal femoral nail or intramedullary hip screw) [1-4]. Reduction is usually achieved by positioning the patient on a fracture table with the foot secured to a boot to aid in traction and rotation. These fractures and positioning for their surgical treatment pose a difficult problem when encountered in patients with below-knee amputations. Absence of the foot and part of the leg in these patients makes positioning on the fracture table challenging. We highlight the difficulties encountered in a patient with bilateral below-knee amputations undergoing fixation of an inter-trochanteric fracture and the various techniques available to overcome this problem.

## Case presentation

A 73-year-old man presented to our department with a history of fall. He complained of pain in the right hip especially on movement of his hip. He had bilateral below-knee amputations following peripheral vascular disease and had below-knee suction prostheses fitted to his lower limbs for mobility. Radiographs of his pelvis and right hip revealed an undisplaced inter-trochanteric fracture of the femur. A dynamic hip screw fixation was planned for the fracture but the dilemma was how to position the patient on the fracture table for the surgery.

**Figure 1:**
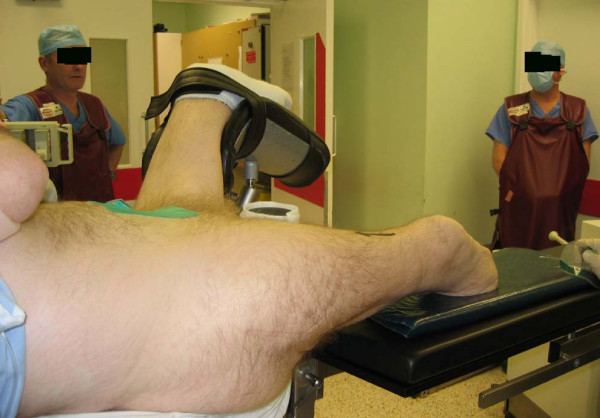
Limb placed on radiolucent leg support with unaffected limb abducted for easy access of the image intensifier.

The patient was positioned on a fracture table with a perineal post and the affected limb supported on a radiolucent leg support (Figure 1). The opposite below-knee stump was strapped securely to a leg support with the limb placed in abduction to allow easy access for the image intensifier (Figure 1). As the fracture was undisplaced, fixation of the fracture was performed with rotation of the hip by the assistant. The procedure was completed satisfactorily and postoperatively the patient was mobile with full weight-bearing after fitting prostheses to his lower limbs.

## Discussion

Inter-trochanteric fractures of the femur are quite common in the elderly. Management of these fractures is essentially surgical and the various techniques used include dynamic hip screw fixation, intramedullary nailing and dynamic condylar screw fixation [1-4]. Patients with bilateral below-knee amputations with inter-trochanteric fractures pose a special problem as positioning them on the fracture table is difficult due to the absence of the foot and part of the leg. The problem is accentuated when there is a need to apply traction for obtaining reduction of the fracture. There is little information in the literature on techniques to deal with this problem. We describe a few methods that can be used when this rare and unusual problem is encountered.

**Figure 2:**
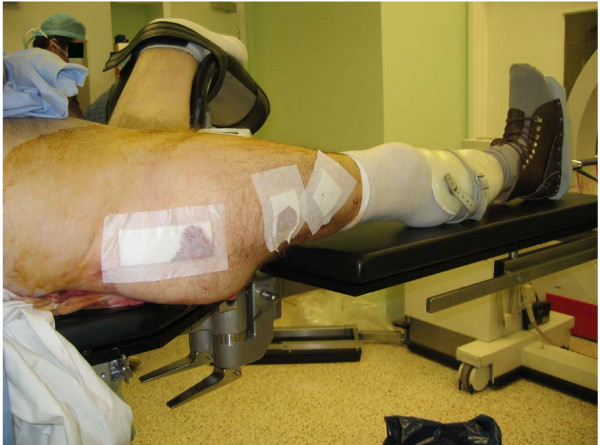
Prosthesis fitted onto the stump and the limb secured on the boot of the traction table.

If the fracture is undisplaced or minimally displaced, the limb can be placed on a radiolucent leg support (Figure 1) with the opposite hip kept abducted to allow access for the image intensifier. Traction and rotation of the hip can be performed by an assistant. An alternative is to fit the patient's prosthesis onto the stump and secure the foot of the prosthesis to the boot on the traction table (Figure 2). A radiolucent leg support should be placed under the limb for safety. These techniques cannot be used when the fracture is displaced and more traction is needed.

**Figure 3:**
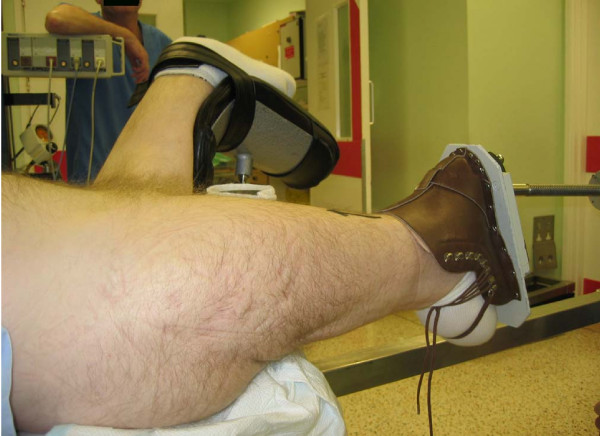
Boot piece inverted to accommodate the flexed knee of the stump.

If the fracture is displaced and greater traction is anticipated, the method of shortening the traction arm and inverting the boot to accommodate the flexed knee (Figure 3) and stump, as described by Al-Harthy *et al. *[5], can be used. A standard boot should be used and the stump should be 12 cm or more (below the tibial tuberosity). If the stump is long, the boot tongue can be inverted for the stump to protrude. Upper tibial skeletal traction can be used if the stump is short but this method has some drawbacks. The skeletal pins may 'cut out' of the bone, which is usually osteoporotic, on applying traction. The other option is to use a distal femoral skeletal traction which would assist in traction.

## Conclusion

Hip fracture fixation surgery in patients with below-knee amputations is a difficult and challenging problem for the surgeon. The dilemma is on how to provide the traction and rotation required for reduction of the fracture. We believe that the techniques mentioned here to overcome this problem are safe and give the surgeon various options to handle this situation.

## Competing interests

The authors declare that they have no competing interests.

## Consent

Written informed consent was obtained from the patient for publication of this case report and any accompanying images. A copy of the written consent is available for review by the Editor-in-Chief of this journal.

## Authors' contributions

UR was involved in collecting patient details, reviewing the literature and drafted the manuscript as the main author. RSY was involved in reviewing the literature and proofreading of the manuscript. AS was involved in critically revising the manuscript for important intellectual content. TKR was involved in conception of the study and revising the manuscript. All authors have read and approved the final manuscript.
